# Activity Patterns and Predator–Prey Temporal Overlap in the High Tiger‐Density Area Bardia National Park, Nepal

**DOI:** 10.1002/ece3.73675

**Published:** 2026-05-21

**Authors:** Bishnu Prasad Shrestha, Joost De Jong, Anouschka R. Hof, Naresh Subedi, Arjun Bhusal, Frank Van Langevelde

**Affiliations:** ^1^ Wildlife Ecology and Conservation Group Wageningen University Wageningen the Netherlands; ^2^ Department of National Parks and Wildlife Conservation Kathmandu Nepal; ^3^ National Trust for Nature Conservation Lalitpur Nepal; ^4^ Zoological Society of London Kathmandu Nepal

**Keywords:** activity pattern, camera trap, predator–prey, temporal overlap

## Abstract

Prey activity patterns are believed to be influenced by, among others, daily and seasonal weather conditions and predation risk. Similarly, predator activities may also be influenced by the same daily and seasonal weather conditions and behavior of prey. Yet there is little research on activity patterns and temporal overlap of predators and prey in tropical regions with strong seasonality and high predator density. To (1) assess the temporal overlap in activity patterns between an apex predator and its main prey in an area of high predator density, and (2) examine the effects of seasonality and prey body size on this overlap, we analyzed data from a 5‐year camera trap survey. We estimated daily activity patterns and assessed temporal overlap between the Royal Bengal tiger ( *Panthera tigris tigris* ), an apex predator, and their primary prey: chital ( *Axis axis* ), sambar ( *Rusa unicolor* ), muntjac (*Muntiacus muntjac*), and hog deer (*Axis porcinus*) *in* Bardia National Park (BNP), a protected area in Nepal with a high density of tigers. We collected camera trap data from May 2019 to April 2024 using 50 camera traps deployed vertically in a 1.4 km × 1.4 km grid in BNP. Our findings indicate that tigers and sambar showed cathemeral activity patterns, whereas hog deer exhibited diurnal patterns, and muntjac and chital displayed crepuscular patterns. Both tigers and deer species, except hog deer, reduced their activity during the hottest parts of the day. There was substantial overlap in activity patterns between tigers and deer, which presents challenges for deer in avoiding predation. This lack of temporal segregation is likely driven by high tiger density. The activity patterns of tigers were consistent across the seasons, whilst those of the deer species were not, suggesting that predator–prey interactions are shaped by ecological factors as well as seasonal weather conditions. These insights of tiger‐prey interactions have management implications for the conservation of tigers in Nepal.

## Graphical abstract

We estimated daily activity patterns and assessed temporal overlap between the Royal Bengal tiger ( *Panthera tigris tigris* ), an apex predator, and their primary prey: chital ( *Axis axis* ), sambar ( *Rusa unicolor* ), muntjac (*Muntiacus muntjac*), and hog deer (*Axis porcinus*) *in* Bardia National Park (BNP), a protected area in Nepal with a high density of tigers. There was substantial overlap in activity patterns between tigers and deer, which presents challenges for deer in avoiding predation. This lack of temporal segregation is likely driven by high tiger density. The activity patterns of tigers were consistent across seasons, whilst those of the deer species were not, suggesting that predator–prey interactions are shaped by ecological factors as well as seasonal weather conditions.

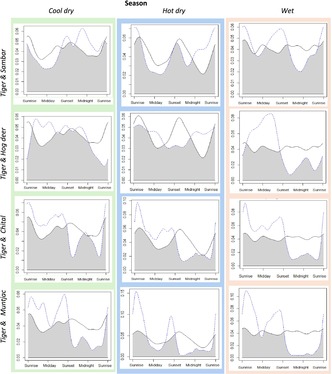

## 1 Introduction

Predator–prey dynamics are primarily shaped by the predator's hunting capabilities and the prey's avoidance strategies. Predators often adjust their behavior to increase the likelihood of encountering prey (Harmsen et al. 2011; Foster et al. 2013). These adjustments result in activity patterns of predators that tend to align with those of prey (Eccard et al. 2008; Gliwicz and Dabrowski 2008). At the same time, prey adapt their behavior accordingly, for example by altering their movement, increasing vigilance, or avoiding high‐risk areas altogether to reduce predation risks (Brown et al. 1999; Laundre et al. 2001). Predation risks may fluctuate during the day and over seasons, corresponding with, among others, changes in weather, predator activity, and hunting success (Sih et al. 2000; Creel et al. 2008; Palmer et al. 2017; Higdon et al. 2019). As a result, prey may adjust the timing of their activities rather than their location to reduce predation risk (Creel et al. 2008). The extent of these behavioral shifts may depend on both prey body size and predator identity, as vulnerability to predation varies among prey species, and predators often target specific prey size classes (Sinclair et al. 2003; Shultz and Finlayson 2010; Preisser and Orrock 2012; Hayward and Kerley 2005; Owen‐Smith and Mills 2008).

Both predators and prey may reduce their activity, typically during the hottest parts of the day, especially during the hot season (Vidal et al. 2011; de Matos Dias et al. 2018; Peksa and Ciach 2018; Berry et al. 2023) and increase their activity towards nighttime (Brivio et al. 2024). During warmer months, activity peaks shift earlier in the morning and later in the evening, as most large mammals have a low heat tolerance (Peterson et al. 2021; Brivio et al. 2024). This may be especially relevant for large‐bodied species that tend to be more vulnerable to heat stress than small‐bodied species (Shrestha et al. 2014). As temperatures and solar radiation increase in summer, daytime activity generally declines (Bourgoin et al. 2011; Brivio et al. 2024). The combined effects of weather and predation are known to significantly influence herbivore movement (Veldhuis et al. 2019, 2020). In response, prey species tend to shift their activity periods to avoid extreme temperatures and reduce predation risk (Veldhuis et al. 2019, 2020).

Understanding how interacting biotic and abiotic pressures shape the activity patterns of prey species is especially important in ecosystems with high density of predators, as prey may be unable to simultaneously cope with both extreme temperatures and predation risk. One area that experiences extreme temperatures and has a high predator (Royal Bengal tiger, *Panthera tigris tigris* ) density is Bardia National Park (BNP) in Nepal (Upadhyaya et al. 2018; DNPWC and DFSC 2022; Shah et al. 2024). Tigers, as apex predators, can play a significant role in structuring terrestrial food webs and are generally regarded as indicators of ecosystem health (Karanth 2003; Dinerstein et al. 2007; Seidensticker 2010; Harihar and Pandav 2012; Carter et al. 2020). Several factors influence the activity patterns of both tigers and their prey (deer species), including predation risk and prey availability, daily and seasonal weather conditions, and human disturbance (Karanth and Sunquist 2000; Maharjan et al. 2025). However, despite their high ecological importance, there are few studies on the daily activity pattern of tigers and their prey, particularly in high tiger density areas (Ramesh et al. 2012; Karanth et al. 2017) and none consider potential differences between seasons. The combined effect of extreme temperatures and high predation risk on the spatiotemporal activity patterns of prey therefore remains unknown. In landscapes with high tiger density, tigers may face increased competition as well as thermal constraints, while prey species experience both high predation pressure as well as thermal constraints. These thermal constraints are likely to be especially pronounced in hotter seasons on the verge of changing global climate (Hetem et al. 2016). To address this knowledge gap, we investigated the seasonal activity patterns of Royal Bengal tigers and their primary prey, the deer species‐ chital ( *Axis axis* ), sambar ( *Rusa unicolor* ), muntjac (*Muntiacus muntjac*), and hog deer ( *Axis porcinus* ) in BNP. Using a multi‐year vertical camera trapping network (He et al. 2025), we assessed how both tigers and their main prey, respond to interacting pressures of temperature and increased competition, predation pressure, and how these factors shape their daily activity patterns across the different seasons. We hypothesized that (i) tigers have a high overlap in activity pattern with their prey, especially the large‐sized sambar, and prey adjust their activity patterns to reduce temporal overlap with tigers (Karanth and Sunquist 1995; Karanth and Sunquist 2000; Ramesh et al. 2012), (ii) the activity pattern of tiger and prey is not constant across the seasons (Ramesh et al. 2012).

## 2 Materials and Methods

### 2.1 Study Area

**FIGURE 1:**
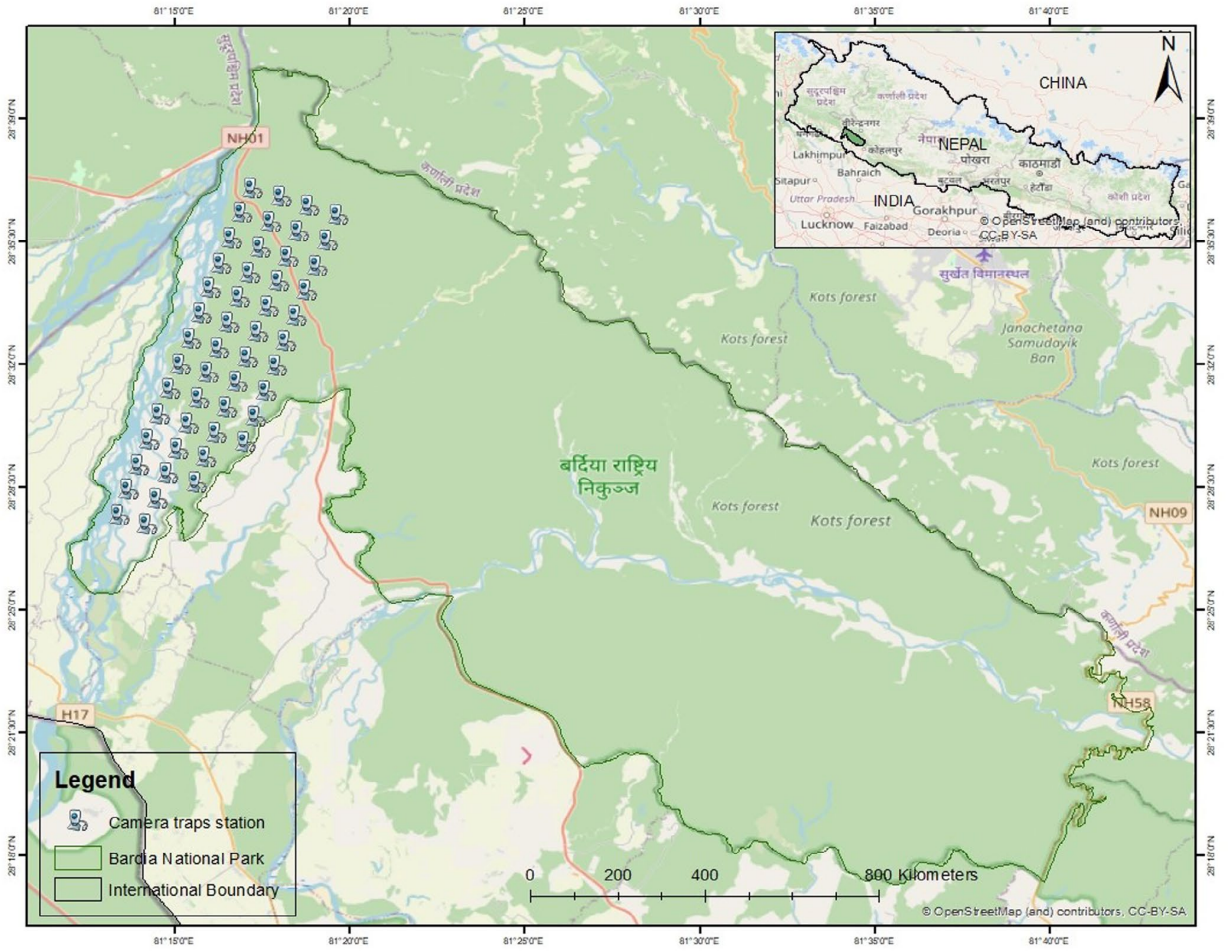
Map showing the study area and camera trap locations.

This study was conducted in Bardia National Park (BNP), Nepal. It lies at coordinates 28°23′0″ N, 81°30′0″ E, spans an area of 968 km^2^ (BNP 2022), and is located in south western Nepal (Figure 1). BNP is well known for its rich biodiversity with 513 species of birds and 62 species of mammals including the Royal Bengal tiger, Asian wild elephant (*Elephus maximus*), Greater one‐horned rhinoceros ( *Rhinoceros unicornis* ), Gangetic dolphin ( *Platanista gangetica* ) (BNP 2022). This park has a high density of tigers, 7.15 tigers per 100 km^2^ (DNPWC and DFSC 2022; Shah et al. 2024). Chital, sambar, muntjac, and hog deer are the major prey of tigers in BNP (Upadhyaya et al. 2018; DNPWC and DFSC 2022; Shah et al. 2024). The combined prey density is approximately 90 individuals per 1 km^2^ (DNPWC and DFSC 2022). Sambar is a large‐sized prey, and spotted deer, muntjac and hog deer are small–midsized prey species in BNP. Chital is the most abundant prey in BNP (Upadhyaya et al. 2018; DNPWC and DFSC 2022).

**FIGURE 2:**
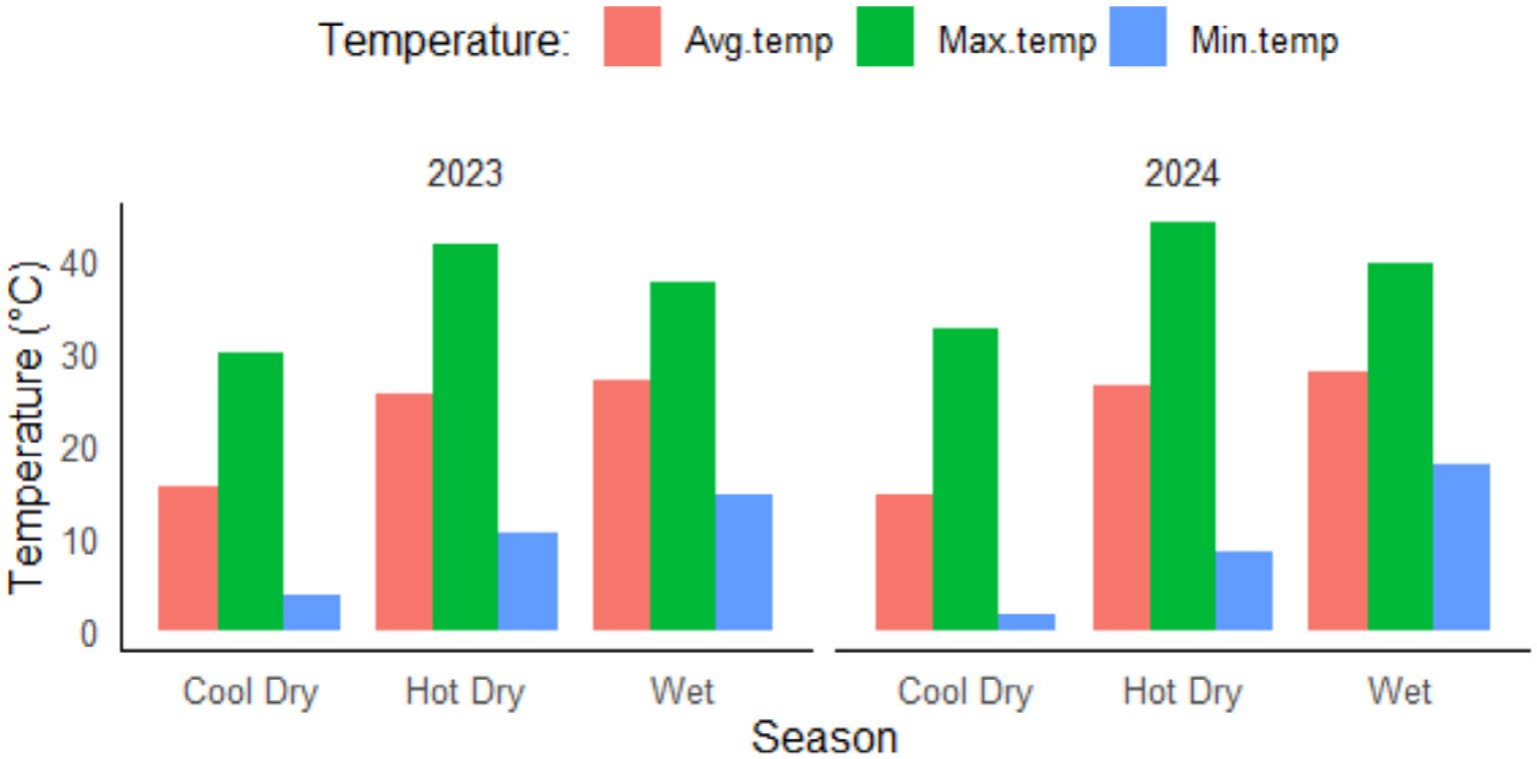
Temperature across different seasons in 2023 and 2024 in BNP (Cool dry season: November–February, Hot dry season: March–June, Wet season: July–October). Avg.temp, average temperature; Max.temp, maximum temperature; Min.temp, minimum temperature.

The park experiences a tropical climate, with a hot season from March to June and a monsoon season from July to October, bringing an average annual rainfall of approximately 1800 mm. Temperatures exceeding 35°C are increasingly common during the hot months (BNP 2022). In 2023 and 2024, the maximum temperatures during the hot dry season (March–June) reached 41.6°C and 43.9°C, respectively (Source: local weather station established in BNP). Usually, temperature reaches a high level around midday between 12:00 and 15:00 during the hot dry season. The average temperatures recorded during the wet season (July–October) were 27.0°C in 2023 and 27.9°C in 2024. Meanwhile, the minimum temperatures during the cool dry season (November–February) dropped to 3.8°C in 2023 and 1.8°C in 2024 (Figure 2). BNP is primarily covered by Sal forest ( *Shorea robusta* ), but it also features a mosaic of other habitats, including grasslands, alluvial plains, fixed forests, and riverine forests (BNP 2022).

### 2.2 Data Collection

Camera trapping has been widely used to study activity patterns of species (Linkie and Ridout 2011; Ramesh et al. 2012; Yang et al. 2019; Allen et al. 2021; Widodo et al. 2022). For this study, camera trap data for tiger and deer species were collected in BNP from May 2019 to April 2024. A total of 50 camera traps were deployed across the Karnali floodplain (Figure 1). Browning BTC‐5HDPX, Reconyx HC500 (HYPERFIRE), and Reconyx HYPERFIRE 2 cameras were used in this study. The cameras were arranged using a 1.4 km × 1.4 km grid system and were equipped with passive infrared motion sensors and infrared illuminators for night‐time imaging (He et al. 2025). Each camera was mounted at a height of 7–8 m in a tree and vertically positioned. The traps operated continuously, day and night, with a 1‐s delay between triggers and were set to capture a maximum of eight photographs per activation. Cameras were checked monthly, and memory cards were replaced during every visit to ensure consistent data collection. All the images of the observations captured in camera traps were stored in Agouti (Casaer et al. 2019): a platform for organizing, processing, standardizing, storing and archiving the camera‐trap images (www.agouti.eu). All the images of animals captured were identified and interpreted, combined with manual annotation and validation (He et al. 2025). Further, a local weather station was established in BNP to measure the temperature at an interval of 15 min.

### 2.3 Activity Pattern and Overlap Analysis

To analyze the daily activity patterns of tiger and the deer species, individual photos were grouped into events by applying a 30 min cut off value. This means a new event started when the time difference between subsequent photos of the same species was more than 30 min. The remaining data were treated as a random sample representing the underlying probability distribution of photographic captures (independent events) across the 24‐h day. This probability density function was interpreted as the species' activity pattern, under the assumption that animals are equally likely to be photographed at any time when active (Ridout and Linkie 2009). For seasonal analysis, the dataset was divided into three distinct seasons: the cool dry season (November to February), the hot dry season (March to June), and the wet/monsoon season (July to October) (Figure 2).

Prior to the daily activity analysis, we converted clock time of independent events to sun time (relative to sunrise and sunset) using the *sunlightTime* function in the overlap R package (Meredith et al. 2024), following Nouvellet et al. (2012). Activity patterns of tigers and the deer species were estimated using non‐parametric kernel density estimation, executed through the overlap package (Ridout and Linkie 2009; Linkie and Ridout 2011) in R version 4.4.3 (R Core Team 2024). Activity levels were plotted against time of day and season to illustrate daily and seasonal activity trends (Ridout and Linkie 2009). Seasonal classification was done using the mutate() function and the case_when() function to categorize the data into these season labels in R. The time was converted into radians (radian_time) to calculate the time of observation in hours (Rowcliffe et al. 2014). The fitact() function from the R activity package was used to fit a model to the circular activity data (Rowcliffe et al. 2014), with 10,000 repetitions of bootstrapping to estimate the activity distribution. The uniformity of the circular distribution of the events collected during the study period was verified using Rayleigh's test (*z*). The Mardia–Watson–Wheeler test was carried out to test the seasonal variation in activity pattern of each species (de Matos Dias et al. 2018).

To quantify the degree of temporal overlap between the tiger and each of the four deer species, the coefficient of overlap ($\hat{\Delta }$)—which ranges from 0 (no overlap) to 1 (complete overlap) was calculated using the overlap package (Meredith and Ridout 2014) in R. Activity overlap is considered “very high” when $\hat{\Delta }$ > 0.9, “high” when 0.75 ≥ $\hat{\Delta }$ ≤ 0.9, “moderate” when 0.5 ≥ $\hat{\Delta }$ ≤ 0.75 and “low” when $\hat{\Delta }$ < 50 (Monterroso et al. 2014). The $\hat{\Delta }4$ estimator was applied for comparisons with more than 75 observations, and the $\hat{\Delta }1$ estimator was used when the sample size was below 75 (Linkie and Ridout 2011). The overlapEst function was used to estimate overlap, and 95% basic bootstrap confidence intervals were generated by bootstrapping 10,000 replicates using the bootCI function (Meredith and Ridout 2014). All data analyses and visualizations were done with R version 4.4.3 (R Core Team 2024).

## 3 Results

We obtained 26,057 independent events of the five target species: tiger (787), sambar (1029), hog deer (358), chital (22,813), and muntjac (1070) (Table 1).

**TABLE 1:** Activity of tiger ( *Panthera tigris tigris* ), sambar ( *Rusa unicolor* ), hog deer ( *Axis porcinus* ), chital ( *Axis axis* ), and muntjac (*Muntiacus muntjac*) across different seasons (Cool dry season: November–February, Hot dry season: March–June, Wet season: July–October).

Season	Sample size	*W*‐statistic	Adjusted *p*
**Cool dry vs. hot dry**			
Tiger	353, 178	0.54	1.000
Sambar	490, 377	15.16	**0.002**
Hog deer	154, 132	11.74	**0.008**
Chital	9406, 6180	88.99	**< 0.001**
Muntjac	519, 272	28.90	**< 0.001**
**Cool dry vs. wet**			
Tiger	353, 256	0.39	1.000
Sambar	490, 162	26.85	**< 0.001**
Hog deer	154, 72	10.03	**0.02**
Chital	9406, 7227	146.87	**< 0.001**
Muntjac	519, 279	6.14	0.14
**Hot dry vs. wet**			
Tiger	178, 256	0.58	1.00
Sambar	377, 162	16.02	**< 0.001**
Hog deer	132, 72	15.04	**0.002**
Chital	6180, 7227	43.30	**< 0.001**
Muntjac	272, 279	21.37	**< 0.001**

*Note:* Bold values show the significant differences.

### 3.1 Activity Pattern of Tiger, Sambar, Hog Deer, Chital and Muntjac Across the Seasons

**FIGURE 3:**
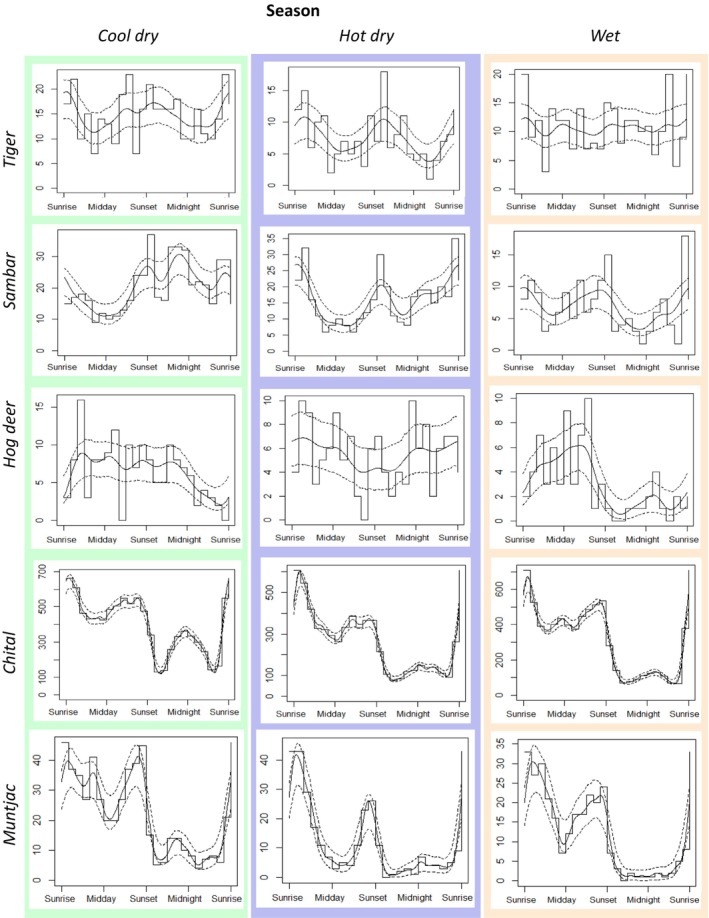
Activity pattern of tiger ( *Panthera tigris tigris* ), sambar ( *Rusa unicolor* ), hog deer ( *Axis porcinus* ), chital ( *Axis axis* ), and muntjac (*Muntiacus muntjac*) across different seasons (Cool dry season: November–February, Hot dry season: March–June, Wet season: July–October).

Tigers exhibited a cathemeral activity pattern, being active throughout the 24‐h period in all seasons. Activity of tigers peaked around sunrise and sunset, and additional activity at night during the cool dry season. During the hot dry season, tigers' activity peaked slightly later after sunrise and again between sunset and midnight, and they reduced their activity during the hottest parts of the day. During the wet season, there was no strong peak of activity, compared to the other seasons (Figure 3). There were no significant differences in activity patterns of tigers between the cool dry and the hot dry season, between the cool dry and the wet season, and between the hot dry and the wet season (Table 1). Similar to tigers, sambar were also cathemeral, active both day and night. They had peaks of activity around sunrise and between sunset and midnight during the cool dry season, while activity peaked around sunrise and sunset during the hot dry and wet season (Figure 3). Like tigers, sambar reduced their activity during the hottest parts of the day during the hot dry season. There were significant differences (*p* < 0.05) in activity patterns of sambar between the seasons (Table 1).

Hog deer displayed a diurnal activity pattern. Activity of hog deer peaked during daytime and dropped after midnight during the cool dry season. In contrast, they were active across the entire 24‐h period during the hot dry season. During the wet season, they were predominantly active during the day and had a peak in their activity between midday and sunset (Figure 3). There were significant differences (*p* < 0.05) in activity pattern of hog deer between seasons (Table 1).

Further, chital and muntjac showed crepuscular activity patterns, with peaks around dawn and dusk that fluctuated seasonally. Both species reduced their activities during the hottest parts of the day in the hot season (Figure 3). There were significant differences (*p* < 0.05) in activity patterns of chital between seasons. There were significant differences (*p* < 0.05) in activity patterns of muntjac between the cool dry and the hot dry season, and between the hot dry and the wet season, but there was no significant difference between the cool dry and the wet season (Table 1).

### 3.2 Activity Overlap Between Tiger and Deer Across Different Seasons

**FIGURE 4:**
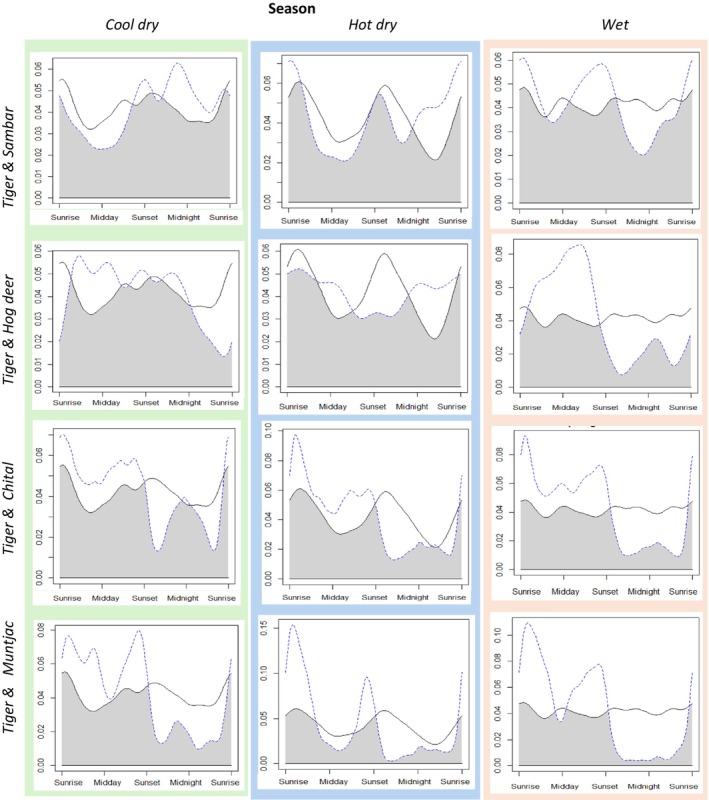
Activity patterns of tigers (solid black line) and deer (sambar, hog deer, chital and muntjac) (dashed blue line), and activity overlaps (gray area) between tigers and deer across different seasons (Cool dry season: November–February, Hot dry season: March–June, Wet season: July–October).

Sambar, chital, and hog deer all exhibited a high degree of temporal overlap with tigers during the cool dry and the hot dry season, with sambar exhibiting the highest overlap during cool dry season (0.88, 0.85 and 0.84 respectively) and hog deer showing the highest overlap during hot dry season (0.85, 0.84 and 0.78 respectively) (Figure 4, Table 2). Muntjac had a moderate temporal overlap with tigers during both the cool dry and the hot dry season (cool dry: 0.7, hot dry: 0.65) (Figure 4, Table 2). During the wet season, sambar exhibited a high degree (0.86) of overlap with tigers whilst hog deer, chital, and muntjac showed a moderate degree of overlap with tigers (0.68, 0.71, and 0.61 respectively).

**TABLE 2:** Activity overlap of tigers and deer (sambar, hog deer, chital, and muntjac) across different seasons (Cool dry season: November–February, Hot dry season: March–June, Wet season: July–October).

Season	Sample size	Overlap coefficient ($\hat{\Delta }4$)	Lower 95% CI	Upper 95% CI	*W*	Adjusted *p*
**Cool dry season**						
Tiger and sambar	353, 490	0.88	0.82	0.94	13.94	**0.011**
Tiger and Hog deer	353, 154	0.85	0.78	0.92	7.05	0.354
Tiger and chital	353, 9406	0.84	0.80	0.88	27.89	**< 0.001**
Tiger and muntjac	353, 519	0.74	0.68	0.80	47.20	**< 0.001**
**Hot dry season**						
Tiger and sambar	178, 377	0.84	0.77	0.91	11.49	**0.038**
Tiger and hog deer	178, 132	0.85	0.77	0.93	2.27	1.000
Tiger and chital	178, 6180	0.78	0.72	0.84	22.16	**< 0.001**
Tiger and muntjac	178, 272	0.65	0.56	0.74	24.74	**< 0.001**
**Wet season**						
Tiger and sambar	256, 162	0.86	0.79	0.93	3.36	1.000
Tiger and hog deer	256, 72	0.68	0.56	0.80	22.36	**< 0.001**
Tiger and chital	256, 7227	0.71	0.64	0.78	49.65	**< 0.001**
Tiger and muntjac	256, 279	0.61	0.52	0.70	53.80	**< 0.001**

*Note:* Bold values show the significant differences.

There were significant differences (*p* < 0.05) in activity patterns of tiger and sambar in the cool dry and hot dry seasons but there was no significant difference in the wet season (Table 2). Contrary, there were no significant differences in activity patterns of tiger and hog deer in the cool dry and hot dry seasons but there was a significant difference (*p* < 0.05) in the wet season (Table 2). There were significant differences (*p* < 0.05) in activity patterns of tiger and chital and of tiger and muntjac across all seasons (Table 2).

## 4 Discussion

Understanding daily activity patterns and the seasonal variation therein of predators and prey is an important component of ecology and conservation, yet knowledge gaps on behavioral responses of both predators and prey to interacting stressors such as thermal constraints and inter−/intraspecific interactions (competition or predation pressures) still exist. Currently, there is especially a lack of understanding how activity patterns vary across seasons and how the density of predators plays a role. Our results shed light on the seasonal variation in the daily activity patterns of an apex predator and its main prey in an area with a high density of predators. We analyzed the activity patterns and temporal overlap of tigers and their four primary prey species (sambar, hog deer, chital, and muntjac) across seasons.

Our results show that tigers were cathemeral, meaning that they were active throughout the 24‐h cycle. These findings are in agreement with a study carried out in Mudumalai Tiger Reserve, India, where tiger density is also high (Ramesh et al. 2012), but in contrast to many other studies that showed tigers were either diurnal, crepuscular, or nocturnal (Linkie and Ridout 2011; Karanth et al. 2017; Yang et al. 2019; Lamichhane et al. 2019; Allen et al. 2020, 2021; Vinitpornsawan and Fuller 2020; Phumanee et al. 2021; Shameer et al. 2021; Chatterjee et al. 2023; Dendup et al. 2023; Maharjan et al. 2025) (Table 3). The main reason for the observed dissimilarities between our and Ramesh et al.'s (2012) study and the others could be the density of tigers. Furthermore, the time of data collection (i.e., seasonality), variation in individual tiger behavior due to intraspecific competition, or varying degrees of interactions with prey species may also play a role. Studies in which a diurnal or nocturnal activity pattern was found, and the tiger density was known, were all conducted in areas with a lower density of tigers: 0.26 to 3.38/100 km^2^ (Lamichhane et al. 2019; Phumanee et al. 2021; Dendup et al. 2023; Maharjan et al. 2025) except for the study by Karanth et al. (2017) in comparison to 7.15/100 km^2^ in our study area (DNPWC and DFSC 2022). The study by Karanth et al. (2017), was however limited to parts of the cool dry season and the hot dry season (Table 3). Whilst there were some peaks of tiger activity during the cool and hot dry season in our study, there was no detectable peak of activity at all during the wet season. The omission of the wet season in the study by Karanth et al. (2017) may therefore explain the differences between our and their findings to some extent.

**TABLE 3:** Our and previous studies on activity pattern of tigers, and temporal overlap with their prey.

Study sites	Activity pattern of tigers	Tiger density (number/100 km^2^)	Study period	Activity overlap with prey	Sources
Bardia National park, Nepal	Cathemeral	7.15	11 May 2019 to 29 April 2024	Cool dry season: sambar (0.89), chital (0.84), hog deer (0.83), muntjac (0.75), hot dry season: sambar (0.85), chital (0.77), hog deer (0.84), muntjac (0.65), wet season: sambar (0.88), chital (0.73), hog deer (0.74), muntjac (0.66)	This work
Parsa National Park, Nepal	Crepuscular	1.74	Dec 2021 to Feb 2022	Gaur (0.72), sambar (0.67), chital (0.61)	Maharjan et al. (2025); DNPWC and DFSC (2022)
Pench Tiger Reserve, India	Crepuscular	4.8 to 5.7	Two period of each year (Nov to Jan; Jan to Mar) from 2013 to 2016	Unknown	Chatterjee et al. (2023)
Jigme Dorji National Park, Bhutan	Crepuscular	0.263	Oct 2021–Jan 2022	Himalayan serow (0.73), musk deer (0.69), muntjac (0.63), sambar (0.68)	Dendup et al. (2023)
Bukit Barisan Selatan NP, Indonesia	Diurnal	Unknown	April–July of each year during 2010–2016	Wild boar (0.8), sambar (0.70), red muntjac (0.68), mouse deer (0.62)	Allen et al. (2021)
Mae Wong and Khlong Lan NPs, Thailand	Diurnal	0.36	Dec 2015 to Sep 2016	Unknown	Phumanee et al. (2021)
Periyar Tiger Reserve, India	Crepuscular	Unknown	17 Nov 2016–17 Nov 2017	Gaur (0.87), sambar (0.83), muntjac (0.61), mouse deer (0.51)	Shameer et al. (2021)
Thung Yai Naresuan Wildlife Sanctuary, Thailand	Crepuscular	Unknown	April 2010–Jan 2012	Gaur (0.73), sambar (0.64), muntjac (0.72)	Vinitpornsawan and Fuller (2020)
Chitwan National Park, Nepal	Crepuscular	3.94	18 Feb 2013 to 4 May 2013	Unknown	Lamichhane et al. (2019)
North eastern section of Jilin province, China	Crepuscular and nocturnal	Unknown	Jan 2014–May 2015	Wild boar (0.8), sika deer (0.79), roe deer (0.79)	Yang et al. (2019)
Nagarhole, Bandipur, Biligiri, and Bhadra Tiger Reserve, India	Nocturnal	Nagarhole‐14 Bandipur‐12 Bhadra‐3.4	Jan–June, 2013	Unknown	Karanth et al. (2017)
Umred‐Karhandla Wildlife Sanctuary, Nagpur, India	Nocturnal	Unknown	27 Nov–25 Dec 2011	Unknown	Athreya et al. (2014)
Mudumalai Tiger Reserve, India	Cathemeral	11.7	Nov to April in 2008, 2009 and 2010	Sambar (0.92), gaur (0.86), chital (0.51)	Ramesh et al. (2012)
Kerinci Seblat National Park, Sumatran rainforests	Crepuscular	Unknown	3 Sep–30 Nov 2004; 3 Jan–29 Mar 2005; 16 April–23 Nov 2006; 24 Aug 2006–2 May 2007	Sambar (0.81), muntjac (0.80), tapir (0.52)	Linkie and Ridout (2011)

As hypothesized, tigers had a high temporal overlap in activity with their prey, especially with the largest prey (sambar) and an abundant prey (chital). The high overlap of tigers and sambar across two seasons suggests that sambar consistently had the highest overlap with tigers of all prey species, suggesting that sambar exhibited limited temporal avoidance and potential reliance on alternative antipredator strategies. Studies conducted in different parts of the tiger range, have also shown that tigers have a high temporal overlap with large –sized prey species such as sambar and gaur ( *Bos gaurus* ) and their primary prey (Linkie and Ridout 2011; Ramesh et al. 2012; Vinitpornsawan and Fuller 2020; Allen et al. 2021; Shameer et al. 2021; Widodo et al. 2022; Dendup et al. 2023; Maharjan et al. 2025) (Table 3). This may indicate that tigers adapt their temporal activity pattern to that of large sized prey and that these prey are unable to avoid this overlap. The high overlap with chital may be explained by the fact that it is the most abundant prey in the region. This is in agreement with findings that large felids generally show high temporal overlap with their primary prey across continents. In Africa, lions ( *Panthera leo* ) and leopards ( *Panthera pardus* ) exhibit strong diel overlap with key ungulate prey, while prey species may adjust activity to reduce predation risk (Balme et al. 2007; Hayward and Slotow 2009; Schuette et al. 2013). Similar patterns occur in the Americas, where ( *Panthera onca* ) and pumas ( *Puma concolor* ) show substantial temporal alignment with major prey species, with overlap varying seasonally and in response to prey behavior (Harmsen et al. 2011; Ruth et al. 2011). Comparable patterns have been documented in Europe, where Eurasian lynx ( *Lynx lynx* ) activity closely tracks that of primary prey such as roe deer ( *Capreolus capreolus* ), and prey species exhibit temporal shifts consistent with antipredator strategies (Monterroso et al. 2014; Heurich et al. 2014).

Further, given the cathemeral activity pattern of tigers in BNP, the primary prey of the tiger had a relatively high overlap (0.61–0.88) with tigers in comparison to other studies conducted in areas with a lower density of tigers (Dendup et al. 2023; Maharjan et al. 2025) (Table 2), where overlap coefficients were lower (0.63–0.73 and 0.61–0.72 respectively). A high overlap (0.51–0.92) was however also observed in Mudumalai Tiger Reserve, India, which also has a high tiger density (Ramesh et al. 2012). These combined results suggest that, as hypothesized, in areas that have a high predator density, prey may not be able to temporarily avoid predators, which can lead to relatively high predation risks.

There was no significant effect of seasonality on tiger activity. In contrast, sambar activity patterns were significantly different among seasons, which indicates that the species responds to seasonal environmental changes and has some behavioral plasticity. Similarly, hog deer, chital, and muntjac activity patterns were significantly different between the cool dry and hot dry seasons, as well as between the hot dry and wet seasons. This suggests that seasonality influences the activity patterns of deer to some extent in BNP, partially supporting our hypothesis. Both tigers and deer, except hog deer, responded to high temperatures by reducing their activity during the hottest parts of the day (midday) during the hot dry season, showing behavioral thermoregulation under prolonged heat waves or extreme environmental conditions. This has also been found by other studies (Vidal et al. 2011; Pagon et al. 2013; Shrestha et al. 2014; Peksa and Ciach 2018; Berry et al. 2023). For instance, Roe deer ( *Capreolus capreolus* ) and several African antelope species reduce their activity in warmer seasons compared to colder seasons (Pagon et al. 2013; Shrestha et al. 2014; Berry et al. 2023). Interestingly, hog deer were also active during the hottest parts of the day during the hot dry season, when tiger activity was lowest, suggesting either a high heat tolerance or a temporal avoidance strategy. Overall, it demonstrates that prey use a range of species‐specific strategies to adapt to seasonal environmental changes and reduce predation risk.

### 4.1 Conservation Implications

Our comprehensive analysis of daily activity patterns of tigers and deer species offers insights relevant for evidence‐based wildlife conservation and management. The observed activity overlap between tigers and their primary prey species suggests that activity patterns of predators and their prey may be influenced by ecological factors as well as by seasonal weather conditions. Notably, in contrast to tigers, prey species demonstrated significant shifts in activity patterns across seasons, highlighting potential behavioral responses to environmental stressors. These findings have several conservation implications. Understanding the timing and intensity of wildlife activity can inform the scheduling of anti‐poaching patrols, which could be concentrated during peak activity hours of target species. Similarly, seasonal variations in prey behavior—especially in response to heat stress—indicate the growing influence of climate change, reinforcing the importance of adaptive management strategies that account for changing thermal environments. In conclusion, activity pattern studies can provide a valuable tool for monitoring predator–prey dynamics and informing adaptive wildlife management. With these results, this paper contributes to understanding how daily and seasonal weather conditions and high predator density may relate to predator–prey dynamics.

## Author Contributions

**Bishnu Prasad Shrestha:** conceptualization (lead), data curation (lead), formal analysis (lead), funding acquisition (equal), methodology (lead), project administration (lead), validation (lead), visualization (lead), writing – original draft (lead), writing – review and editing (equal). **Joost De Jong:** conceptualization (equal), methodology (equal), supervision (equal), writing – review and editing (equal). **Anouschka R. Hof:** conceptualization (equal), methodology (equal), supervision (equal), writing – review and editing (equal). **Naresh Subedi:** conceptualization (supporting), methodology (equal), supervision (equal), writing – review and editing (equal). **Arjun Bhusal:** data curation (equal), formal analysis (supporting), validation (equal), visualization (equal). **Frank Van Langevelde:** conceptualization (lead), funding acquisition (lead), methodology (equal), supervision (lead), writing – review and editing (equal).

## Funding

NWO “Save the Tiger! Save the Grasslands! Save the Water!” project, and Himalayan Tiger Foundation (HTF), the Netherlands.

## Conflicts of Interest

The authors declare no conflicts of interest.

## Supporting information


**Data S1:** ece373675‐sup‐0001‐Supinfo1.csv.

## Data Availability

The data file has been provided as a Supporting Information (Shrestha_Supporting_Data.csv). As the original dataset will also be used in our other research projects, only the portion relevant to this study has been included.
